# Salivary epigenetic biomarkers as predictors of emerging childhood obesity

**DOI:** 10.1186/s12881-020-0968-7

**Published:** 2020-02-14

**Authors:** Amanda Rushing, Evan C. Sommer, Shilin Zhao, Eli K. Po’e, Shari L. Barkin

**Affiliations:** 1 Louisiana State University Health Sciences Center, School of Medicine, 1901 Perdido Street, New Orleans, LA 70112 USA; 2 Department of Pediatrics, Vanderbilt University Medical Center, 2146 Belcourt Ave, Nashville, TN 37232-9225 USA; 3 Department of Biostatistics, Vanderbilt University Medical Center, 571 Preston Research Building, 2220 Pierce Ave, Nashville, TN 37232-6838 USA; 4 Department of Pediatrics, Vanderbilt University Medical Center, 2146 Belcourt Ave, Nashville, TN 37232-9225 USA; 5 Department of Pediatrics, Vanderbilt University School of Medicine, 2200 Children’s Way, Doctor’s Office Tower 8232, Nashville, TN 37232-9225 USA

**Keywords:** Obesity, Hispanic children, Epigenetics, Methylation, Saliva

## Abstract

**Background:**

Epigenetics could facilitate greater understanding of disparities in the emergence of childhood obesity. While blood is a common tissue used in human epigenetic studies, saliva is a promising tissue. Our prior findings in non-obese preschool-aged Hispanic children identified 17 CpG dinucleotides for which differential methylation in saliva at baseline was associated with maternal obesity status. The current study investigated to what extent baseline DNA methylation in salivary samples in these 3–5-year-old Hispanic children predicted the incidence of childhood obesity in a 3-year prospective cohort.

**Methods:**

We examined a subsample (*n* = 92) of Growing Right Onto Wellness (GROW) trial participants who were randomly selected at baseline, prior to randomization, based on maternal phenotype (obese or non-obese). Baseline saliva samples were collected using the Oragene DNA saliva kit. Objective data were collected on child height and weight at baseline and 36 months later. Methylation arrays were processed using standard protocol. Associations between child obesity at 36 months and baseline salivary methylation at the previously identified 17 CpG dinucleotides were evaluated using multivariable logistic regression models.

**Results:**

Among the *n* = 75 children eligible for analysis, baseline methylation of Cg1307483 (*NRF*1) was significantly associated with emerging childhood obesity at 36-month follow-up (OR = 2.98, *p* = 0.04), after adjusting for child age, gender, child baseline BMI-Z, and adult baseline BMI. This translates to a model-estimated 48% chance of child obesity at 36-month follow-up for a child at the 75th percentile of *NRF1* baseline methylation versus only a 30% chance of obesity for a similar child at the 25th percentile. Consistent with other studies, a higher baseline child BMI-Z during the preschool period was associated with the emergence of obesity 3 years later, but baseline methylation of *NRF1* was associated with later obesity even after adjusting for child baseline BMI-Z.

**Conclusions:**

Saliva offers a non-invasive means of DNA collection and epigenetic analysis. Our proof of principle study provides sound empirical evidence supporting DNA methylation in salivary tissue as a potential predictor of subsequent childhood obesity for Hispanic children. *NFR1* could be a target for further exploration of obesity in this population.

## Background

The prevalence of pediatric obesity has been increasing at an alarming rate in the last forty years [1, 2]. Although pediatric obesity prevalence is a global issue, the United States is facing epidemic levels of pediatric obesity [3, 4]. The Center for Disease Control and Prevention indicates that the prevalence of obesity among children aged 2–19 years old has risen from 13.9% in 2000 to 18.5% in 2016 [5]. However, some ethnic groups have an even higher obesity prevalence [1, 6]. For example, the 2015–2016 National Health and Nutrition Examination Survey (NHANES) reported 25.8% of Hispanic 2–19 year-olds were obese compared to 14.1% of their non-Hispanic white counterparts [7]. Identifying what influences different populations is critical to successfully reducing obesity-related health disparities.

Childhood is a particularly sensitive period for neurological, endocrine, and metabolic development. For example, obesity at a young age contributes to an increased risk of diabetes, hypertension, and cardiovascular disease in adulthood [8–10]. Recent literature indicates that susceptibility to obesity within an “obesogenic” environment differs among individuals [11, 12]. It is not clear what mechanisms are responsible for obesity variation, but many studies identify a dynamic interaction of genetic and environmental exposures at sensitive periods of development [13, 14]. While monogenic DNA mutations exist and are associated with obesity, common forms of childhood obesity have frustrated the scientific community with the so-called problem of missing heritability. It appears that obesity is a multi-trait, multi-state phenotype. The field of epigenetics, modifications that affect transcriptional plasticity, might offer insights into the emerging phenotype of childhood obesity. Epigenetic patterns, often measured by DNA methylation, change rapidly in response to environmental factors such as nutrition and physical activity and are specifically vulnerable to changes during early childhood development. Moreover, epigenetic patterns vary between ethnic groups and could explain differing susceptibility to early emerging obesity and its commonly associated later chronic diseases [15–18].

Epigenetic patterns are tissue-dependent. While blood is a common tissue used in studies of human epigenetic changes, saliva is also a promising tissue. Saliva could be particularly valuable in studying pediatric populations given the ease of tissue access, cost-efficiency, and the ability to collect it in multiple settings [19, 20]. Abraham and colleagues illustrated that when comparing DNA fragmentation, quality, and genotype concordance, saliva is comparable to blood samples [21]. When examining methylation patterns, both saliva and blood reliably assess epigenetic modifications [22]. In comparing the collection of blood and saliva samples, saliva collection is associated with lower infection rates, decreased cost, increased patient acceptance, and higher participant compliance [23]. Saliva also has the advantage of offering insight into the gastrointestinal tract, which could be useful when examining obesity. The ease of saliva collection coupled with DNA fidelity could allow for a more practical source of DNA collection for children. Given that salivary tissue is used less often for epigenetic studies, this approach is novel.

Recently, Oelsner et al. examined 92 saliva samples from 3 to 5-year-old Hispanic children who were at risk for obesity but not yet obese and analyzed 936 genes previously associated with obesity [24]. The cross-sectional study identified 17 CpG dinucleotides that demonstrated an association between baseline differential child DNA methylation and maternal BMI (obese versus non-obese). While this analysis was conducted on baseline saliva samples, these children subsequently participated in a three-year longitudinal study where more than a third of children became obese. The current study investigates to what extent baseline child salivary DNA methylation patterns were associated with the emerging incidence of childhood obesity in this 3-year prospective cohort of young Hispanic children [25].

## Methods

### Informed consent

Trained, bilingual study staff administered written consents to the parent or legal guardian of the child of interest in the language of their choice (English or Spanish). The parent or legal guardian provided consent for both themselves and their child. Because this was a low health literacy population, the consenting process utilized specific measures to ensure participant understanding including a “teach-back” method and protocol visual aids [26]. The study was approved by Vanderbilt University Review Board (IRB No. 120643).

### Sample population study subjects

We examined a subsample (*n* = 92) of Growing Right Onto Wellness (GROW) trial participants [24]. They were randomly selected at baseline, before randomization, based on maternal phenotype. One group of children had obese mothers (BMI ≥30 and waist circumference ≥ 100 cm), and the other had non-obese mothers (BMI < 30 and waist circumference < 100 cm). The two groups were matched on child age and gender. The current study examines this subsample as a prospective cohort, after a 3-year follow-up.

Child participant eligibility criteria in the original GROW study included: 3–5 years old, no known medical conditions, and being at risk for obesity (high normal weight or overweight) but not yet obese (BMI ≥50th and < 95th percentile). Three children were excluded due to being obese at baseline. Parent eligibility criteria included: ≥18 years old, signed written consent to participate in a 3-year trial, consistent phone access, spoke English or Spanish, and no known medical conditions that would preclude routine physical activity. Families were recruited from East Nashville and South Nashville. All parents self-reported that at least one person in their household was eligible to participate in a program that qualified them as underserved. The underserved programs included but were not limited to TennCare (Medicaid), Special Supplemental Nutrition Program for Women, Infants, and Children (WIC), CoverKids, Food Stamps, and/or reduced-price school meals [25]. To be eligible for analysis in the epigenetic study reported here, child BMI must have been collected at the 36-month follow-up (*n* = 75).

### Saliva collection and assay method

Baseline saliva samples from children were collected voluntarily from interested participants using a separate consenting form in the participant’s language of choice. Saliva was collected from children at baseline using the Oragene DNA saliva kit. Children were asked to fast for 30 min and rinse their mouths with water immediately before collection. Two mL of saliva was collected from children using saliva sponges, inserted between the cheek and gums in the upper cheek pouch without swabbing the buccal mucosa. Samples were collected at home or in community centers. To ensure safety and decrease contamination, trained sample collectors wore gloves and capped the samples as soon as the saliva was collected. After samples were properly collected and labeled, the samples were sent to the Vanderbilt Technology for Advanced Genomics (VANTAGE) core at Vanderbilt University. DNA was then extracted from the saliva using the PrepIT L2P reagent with guidance from DNA Genotek’s recommendations and stored at − 80 degrees Centigrade.

### Anthropometric data

Objective height and weight for parent-child pairs were collected and used to calculate BMI (kg/m^2^) at baseline and 36 months. Trained research staff collected weight and height of participants using standard anthropometric procedures, and participants wore only light clothing and no shoes. Height was measured to the nearest 0.1 cm using a stadiometer (Perspective Enterprise, Portage, MI), and weight was measured to the nearest 0.1 kg using a calibrated scale. BMI measurements were collected using the trial protocol [27]. BMI-Z was calculated based on each child’s BMI, gender, and age, and BMI categories were defined using CDC guidelines: normal weight (<85th percentile); overweight (≥85th and < 95th percentile); and obese (≥95th percentile).

### Statistical analysis

Categorical variables were summarized using frequencies and percentages, and Pearson’s chi-squared test was used to evaluate baseline differences. Continuous variables were summarized using means and standard deviations, and the Wilcoxon rank-sum test was used to evaluate baseline differences.

Genome-wide DNA methylation was conducted on the 92 saliva samples using the Infinium Illumina HumanMethylation 450 K BeadChip (Illumina, San Diego, CA, USA). Methylation arrays were processed using a standard protocol [24, 28] and quality control was done using the Methylation module (V1.9.0). Samples with a call rate lower than 98% were excluded, resulting in the removal of one sample (total baseline eligible sample *n* = 91). The Background Subtraction method [29] was used for methylation array normalization. In this method, the average signals of built-in negative controls represent background noise and are subtracted from all probe signals to make unexpressed targets equal to zero. Outliers were removed using the median absolute deviation method. Lastly, the normalized values were log-transformed and multiplied by 10 to put degree of methylation on a continuous scale from 0 to 10 for statistical analysis.

Associations between child obesity at 36 months and baseline methylation levels were evaluated using multivariable logistic regression models. Other variables were included in the models as covariates, including child age, child baseline BMI-Z, child gender, and parent baseline BMI. Statistical significance was defined as *p* < 0.05. All analyses were performed using R software (www.r-project.org) version 3.5.0.

## Results

Of the original 92 participants in the baseline subsample, 75 met quality control and eligibility requirements and were included for analysis. The mean age was 4.3 years (SD = 0.8), and the mean baseline BMI was 16.7 (SD = 0.8). Within the analytic sample, at baseline, 64.0% (*n* = 48) were normal weight, and 36.0% (*n* = 27) were overweight, and 73.3% (*n* = 55) were Hispanic-Mexican. Among parents, 48.0% (*n* = 36) were obese (stratified by design for this subsample). Refer to Table 1 for further baseline demographic descriptions of the sample. At the study’s conclusion, 37% (*n* = 28) of children were obese.

**Table 1:** Demographics of Sample Population^a^

Child Characteristics	Total (*n* = 75)
Gender	
Male	35 (46.7%)
Female	40 (53.3%)
Age at anthropometry collection (years)	4.3 (0.8)
Age category (years)	
3	34 (45.3%)
4	26 (34.7%)
5	15 (20.0%)
BMI (kg/m^2^)	16.7 (0.8)
BMI-Z	0.9 (0.5)
BMI category	
Normal weight	48 (64.0%)
Overweight	27 (36.0%)
Waist circumference (cm)	53.4 (3.0)
Race/Ethnicity	
Hispanic Mexican	55 (73.3%)
Hispanic non-Mexican	20 (26.7%)
**Parent Characteristics**	
Age (years)	32.0 (5.7)
BMI (kg/m^2^)	29.4 (7.3)
BMI category	
Normal weight	28 (37.3%)
Overweight	11 (14.7%)
Obese	36 (48.0%)
Waist circumference (cm)	97.9 (16.1)

*Abbreviations: BMI* body mass index*, BMI-Z* BMI z-score

^a^ Values are mean (SD) or frequency (percent)

Comparing children who were non-obese at 36 months (*n* = 47) to those who were obese at 36 months (*n* = 28), there were no statistically significant differences in baseline child characteristics, although, descriptively, baseline weight-related characteristics appeared to be lower in children who were not obese at follow-up. Parents did not have any significantly different baseline characteristics between the two groups, although mean age was descriptively slightly younger in parents of children who became obese at 36 months (33.0 vs. 30.4) (Table 2).

**Table 2:** Baseline Participant Characteristics by Child Obesity Status at 36 months^a^

Child Characteristics	Child not obese at 36 months (*n* = 47)	Child obese at 36 months (*n* = 28)	*P* value^b^
Gender			0.32
Male	24 (51.1%)	11 (39.3%)	
Female	23 (48.9%)	17 (60.7%)	
Age at anthropometry collection (years)	4.3 (0.8)	4.3 (0.8)	0.90
Age category (years)			0.93
3	21 (44.7%)	13 (46.4%)	
4	17 (36.2%)	9 (32.1%)	
5	9 (19.1%)	6 (21.4%)	
BMI (kg/m^2^)	16.6 (0.7)	16.9 (0.8)	0.06
BMI-Z	0.8 (0.5)	1.0 (0.4)	0.06
BMI category			0.05
Normal weight	34 (72.3%)	14 (50.0%)	
Overweight	13 (27.7%)	14 (50.0%)	
Waist circumference (cm)	52.9 (2.6)	54.3 (3.6)	0.13
Race/Ethnicity			0.41
Hispanic Mexican	36 (76.6%)	19 (67.9%)	
Hispanic non-Mexican	11 (23.4%)	9 (32.1%)	
**Parent Characteristics**			
Age (years)	33.0 (5.2)	30.4 (6.0)	0.06
BMI (kg/m^2^)	29.2 (7.4)	29.7 (7.3)	0.88
BMI category			0.67
Normal weight	18 (38.3%)	10 (35.7%)	
Overweight	8 (17.0%)	3 (10.7%)	
Obese	21 (44.7%)	15 (53.6%)	
Waist circumference (cm)	97.0 (16.2)	99.3 (16.1)	0.47

*Abbreviations: BMI* body mass index*, BMI-Z* BMI z-score

^a^ Values are mean (SD) or frequency (percent)

^b^ Wilcoxon rank-sum test used for continuous variables, and Pearson’s chi-squared used for categorical variables

Table 3 describes the associations between children’s continuous degree of baseline methylation at each CpG dinucleotide and childhood obesity at 36 months for the 75 children with follow-up data. The multivariable logistic regression model was adjusted for child gender, baseline age, baseline BMI-Z, and adult baseline BMI. After accounting for these covariates, higher baseline methylation of cg01307483 (*NRF*1) was significantly associated with a higher probability of childhood obesity at 36 months (odds ratio = 2.98, 95% CI = [1.06, 8.38], *p* = 0.04). *PPARGC1B* methylation was potentially associated with decreased obesity at 36 months but was not statistically significant. *SORCS2* methylation was not statistically significant for cg03218460 or cg18431297 [30, 31].

**Table 3:** Association of Baseline Methylation^a^ at Each CpG Dinucleotide With Child Obesity Status at 36-Month Follow-Up

Unique CpG Dinucleotide	Associated Gene	Odds Ratio	95% CI	***p***-value	Biological Relevance
cg21790991	*FSTL1*	1.35	[0.88, 2.06]	0.16	Regulate endothelial cell function and vascular remodeling in response to hypoxic ischemia [30, 40]
cg03218460	*SORCS2*	2.08	[0.83, 5.21]	0.12	Functions to regulate fasting insulin levels and secretion of insulin, diabetes susceptibility [41, 42]
cg23241637	*ZNF804A*	1.47	[0.61, 3.49]	0.39	Schizophrenia [43, 44]
cg04798490	*SHANK2*	1.51	[0.6, 3.8]	0.38	Autism [45, 46]
**cg01307483**	***NRF1***	**2.98**	**[1.06, 8.38]**	0.04*	**Innate immune response governing adipocyte inflammation, cytokine expression, and insulin resistance** [34–36]
cg19312314	*CBS*	0.75	[0.27, 2.13]	0.59	Catalyzes the conversion of homocysteine to cystathionine, associated with homocystinuria and hydrogen sulfide production [47]
cg14321859	*DLC1*	1.82	[0.69, 4.81]	0.23	Regulates Rho GTP-binding proteins, cytoskeletal signaling, tumor suppressor, adipocyte differentiation [48, 49]
cg03067613	*ATP8B3*	1.24	[0.4, 3.8]	0.71	Reproduction [37]
cg11296553	*CEP72*	0.99	[0.03, 32.38]	0.99	Ulcerative colitis [38]
cg16509445	*CRYL1*	1.04	[0.23, 4.63]	0.96	Heptocellular carcinoma [50]
cg16344026	*PPARGC1B*	0.27	[0.04, 2.04]	0.21	Fat oxidation, non-oxidative glucose metabolism, and energy regulation, ubiquitous in duodenum and small intestines [51, 52]
cg15354625	*ODZ4*	0.78	[0.12, 4.94]	0.79	Bipolar disorder [53]
cg23836542	*CHN2*	1.13	[0.14, 9.07]	0.91	Encodes GTP-metabolizing protein that regulates cell proliferation and migration, insulin resistance [54, 55]
cg07511564	*NXPH1*	1.02	[0.34, 3.03]	0.97	Forms a tight complex with alpha neurexins, promoting adhesion between dendrites and axons, diabetic neuropathy [56, 57]
cg18799510	*GRIN3A*	1.02	[0.32, 3.28]	0.98	Schizophrenia [31, 58]
cg14996807	*UNC13A*	3.17	[0.64, 15.76]	0.16	ALS [59, 60]
cg18431297	*SORCS2*	1.01	[0.51, 1.99]	0.98	Neuropeptide receptor activity, strongly expressed in the central nervous system, acts with IGF1 in the setting of cardiovascular disease [42, 61]

^a^ Degree of methylation was a continuous variable calculated by log-transforming the normalized values and multiplying by 10 to put it on a scale from 0 to 10 (see Methods section)

**Fig. 1:**
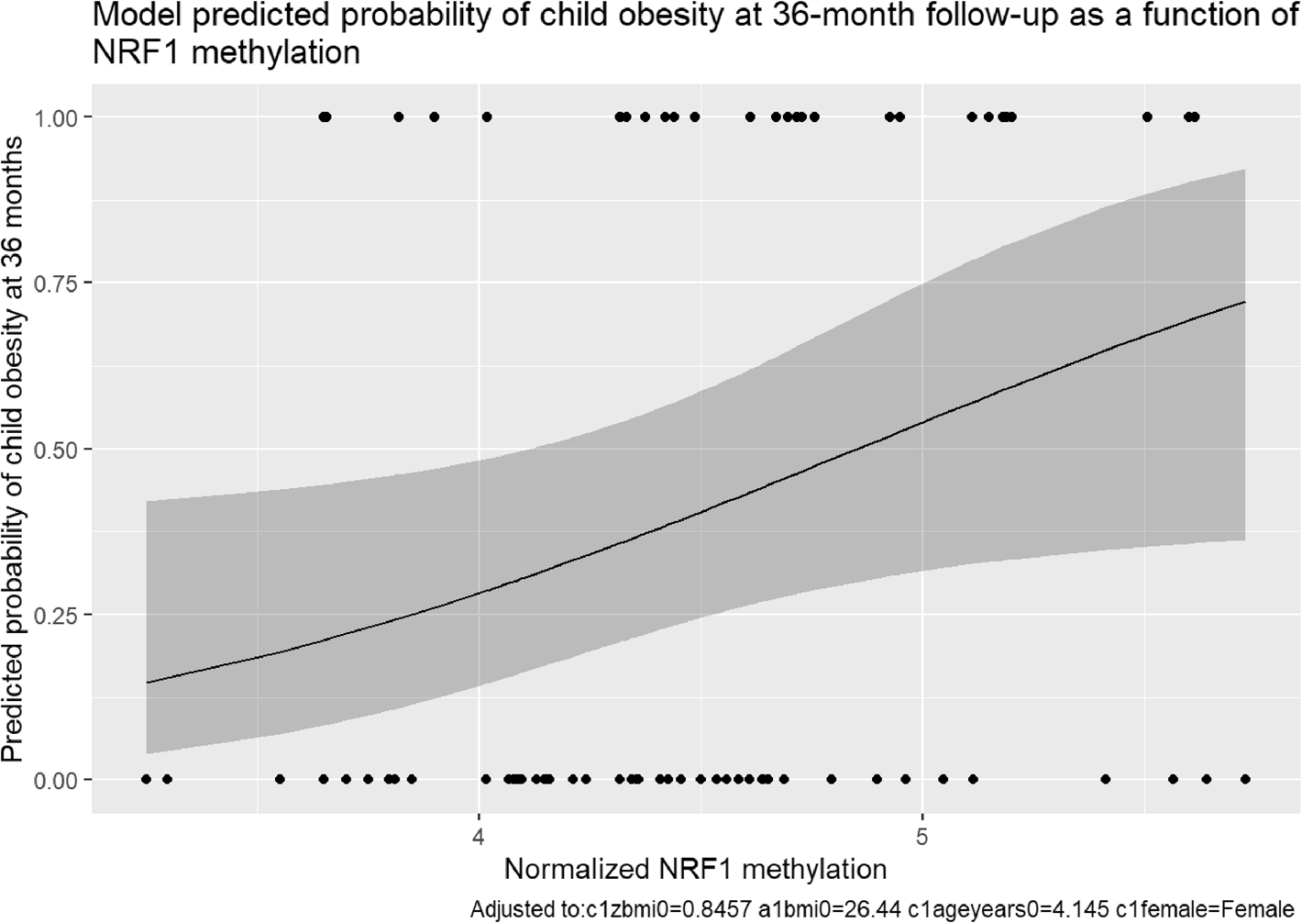
Model Predicted Probability of Child Obesity at 36-month Follow-up as a Function of NRF1 Methylation. Figure 1 displays the logistic regression model-predicted probability of child obesity at 36 months as a function of the degree of methylation of cg01307483 (*NRF*1). The solid line indicates the predicted probability, and the gray shaded region represents the 95% confidence interval. As the degree of methylation of *NRF1* increases, the probability of child obesity at 36 months increases significantly

In the logistic regression model analyzing the *NRF1* dinucleotide, child gender, child baseline age, and baseline parent BMI, were not significant predictors of childhood obesity at 36 months (Table 4). However, in this model, child baseline BMI-Z and baseline differential methylation of *NRF*1 were significant predictors of childhood obesity at 36 months. Figure 1 displays the model-predicted probability of child obesity at 36-month follow-up as a function of increasing *NRF1* methylation. Child baseline BMI-Z was a significant predictor in all but two CpG dinucleotide models (odds ratio = 3.09–4.08, *p* < 0.05). Median degree of baseline methylation at each CpG dinucleotide examined in this study is shown in Additional file 1: Table S1.

**Table 4:** Association of baseline differential DNA methylation^a^ with obesity at 36 months, adjusted for co-variates

	Odds Ratio	95% CI	***P*** Value
**Child**			
Baseline BMI-Z	3.25	[1.00, 10.50]	0.049
Baseline age	1.50	[0.76, 2.94]	0.24
Gender (male)	0.59	[0.21, 1.63]	0.31
**Parent**			
Baseline BMI	0.99	[0.92, 1.07]	0.86
**CpG Baseline Methylation**			
Cg10307483 (*NRF*1)	2.98	[1.06, 8.38]	0.04

^a^ Degree of methylation was a continuous variable calculated by log-transforming the normalized values and multiplying by 10 to put it on a scale from 0 to 10 (see Methods section)

## Discussion

To our knowledge, this is the first prospective cohort study that investigates DNA methylation collected via salivary samples as a predictor of childhood emerging obesity among 3–5-year-old Hispanic children. Although other studies have investigated DNA methylation patterns in children who are already obese, our prospective study investigated how these patterns might be used to predict the future emergence of obesity in non-obese preschool-aged children above and beyond what is provided by their age, gender, baseline BMI-Z, and their mother’s BMI. After adjusting for these covariates, baseline methylation of Cg1307483 (*NRF*1) was significantly associated with emerging childhood obesity at 36-month follow-up with a significant positive odds ratio (OR = 2.98, *p* = 0.04). To place this odds ratio finding into context and enhance interpretation, the model estimated a 48% chance of child obesity at 36-month follow-up for a child at the 75th percentile of *NRF1* methylation versus only a 30% chance of obesity for a similar child at the 25th percentile.

Consistent with other studies, a higher baseline child BMI-Z during the preschool period was associated with the emergence of obesity 3 years later, but baseline methylation of *NRF1* was associated with later obesity even after adjusting for baseline BMI-Z. *NRF*1 is associated with the innate immune response governing adipocyte inflammation and cytokine expression, as well as brown adipose tissue thermogenic adaption. It also plays a role in insulin resistance [34–36]. The current results build on the existing literature by demonstrating that DNA methylation of a critical CpG dinucleotide within the *NRF1* gene in 3–5-year-old non-obese children is associated with the emergence of obesity 3 years later. This provides a potential target of further investigation and suggests that adipocyte inflammation might already be affected before the phenotypic emergence of childhood obesity in Hispanic children. Other studies demonstrate that early life exposures can affect later health and disease outcomes. This life-course understanding of emerging phenotypes might contribute to health disparities.

It is important to note that prior studies implicated *NRF1* associated with existing obesity and young Hispanic children using blood and skeletal muscle samples as well [36]. Comuzzie and colleagues investigated chromosome 7q in Hispanic children and found unique loci contributing to pediatric obesity [33]. As in our study, the genes significantly associated with obesity suggest a strong inflammatory influence. While our study does not confirm this hypothesis, it does provide supplemental data supporting this theory. Because *NRF1* is a major transcription factor in metabolic regulation and stimulates the expression of *PPARGC1B,* these look like promising targets for further research and intervention approaches. Although direct comparisons between saliva and blood cannot be made in the current study, the fact that we noted the same association in saliva as found in blood and skeletal muscle further bolsters support for the use of saliva as a useful tissue for epigenetic inquiry.

Other genes could also play an important role in both predicting later obesity and understanding the pathways that lead to obesity. For example, *PPARGC1B* methylation had a potentially strong association with *decreased* obesity at 36 months but was not statistically significant. *PPARGC1B* is associated with fat oxidation, non-oxidative glucose metabolism, and energy regulation [37, 38]. Similarly, *SORCS2* methylation, which functions to regulate fasting insulin levels and secretion of insulin, was potentially associated with *increased* obesity at 36 months but was not statistically significant in this relatively small sample [31, 39]. Repeating this work in a larger sample is necessary for further understanding these and other epigenetic contributions to the early emergence of obesity in populations who experience higher health disparities associated with obesity. Moreover, while the small analytic sample size precluded moderation analysis in the current study, it would be interesting for future research to explore whether the potential relationships between methylation and subsequent obesity status depend on initial BMI status or other potential moderators of interest (e.g., gender, ethnicity, income, etc.).

To-date, many epigenetic studies have focused on the exploration of molecular pathways. While it is not yet known if these DNA methylation patterns can be used as biomarkers, our study provides a proof-of-principle demonstrating that even in non-obese Hispanic children, some differential methylation patterns are associated with the later emergence of obesity. While it is clear that susceptibility to obesity within an “obesogenic” environment varies among individuals, it is not clear why. This line of epigenetic inquiry using saliva as an accessible tissue for pediatric study holds promise for guiding further exploration in both understanding and intervening before the emergence of childhood obesity.

Although *NFR1* was significantly related to child obesity at 36-month follow-up, the relatively small sample size analyzed in this study might have contributed to a failure to detect important relationships for the other CpG dinucleotides. Expanding the current analysis to include larger sample sizes would help to confirm and validate the findings. While there was a strict collection protocol for saliva collection, contamination and human collection error are possible when collecting salivary DNA. Although previous literature indicates DNA methylation in saliva and blood samples are similar, the current study only investigated methylation patterns in saliva and cannot be used to make direct comparisons to blood. Furthermore, although this sample yields insight into Hispanic 3–5-year-olds, DNA methylation patterns should be studied in children of various ages and race/ethnicities.

## Conclusions

Saliva offers a non-invasive means of DNA collection and epigenetic analysis. This proof of principle study provides empirical evidence supporting the idea that DNA methylation assessed using salivary tissue collected in non-obese children could be used as an important predictor of childhood obesity 3 years later. *NFR1* could be a target for further exploration of obesity in Hispanic children.

## Supplementary information

**Additional file 1: Table S1.** CpG Dinucleotide Methylation.

## Data Availability

The genetic dataset supporting the conclusions of this article are available in NCBI’s Gene Expression Omnibus and are accessible through GEO Series access number GSE72556 (https://www.ncbi.nlm.nih.gov/geo/query/acc.cgi?acc=GSE72556).

## Abbreviations

BMI: Body Mass Index
BMI-Z: Body Mass Index Z-score
DNA: Deoxyribonucleic Acid
GROW: The Growing Right Onto Wellness Trial
NHANES: National Health and Nutrition Examination Survey
VANTAGE: The Vanderbilt Technologies for Advanced Genomics
WIC: The Special Supplemental Nutrition Program for Women, Infants, and Children
