# Supplementary material for: Salivary epigenetic biomarkers as predictors of emerging childhood obesity
Source: BMC Med Genet. 2020 Feb 14;21:34. doi: 10.1186/s12881-020-0968-7 (PMC7023819; doi:10.1186/s12881-020-0968-7)
Supplement: Supplementary file 1 — Additional file 1: Table S1. CpG Dinucleotide Methylation. [file 12881_2020_968_MOESM1_ESM.docx]

Table S1. CpG Dinucleotide Methylation^1^

|  | Child not obese at 36 months (N=47) | Child obese at 36 months (N=28) | Total (N=75) |
| --- | --- | --- | --- |
| **CpG dinucleotide** |  |  |  |
| cg21790991 | 5.5 (5.2, 6.1) | 5.7 (5.1, 6.9) | 5.5 (5.2, 6.6) |
| cg03218460 | 7.2 (6.9, 7.6) | 7.3 (7.1, 7.5) | 7.2 (6.9, 7.6) |
| cg23241637 | 1.6 (1.3, 2.1) | 1.8 (1.4, 2.1) | 1.7 (1.4, 2.1) |
| cg04798490 | 6.0 (5.7, 6.5) | 6.0 (5.8, 6.7) | 6.0 (5.8, 6.6) |
| cg01307483 | 4.3 (4.1, 4.6) | 4.7 (4.3, 5.1) | 4.4 (4.1, 4.8) |
| cg19312314 | 9.5 (9.0, 9.6) | 9.4 (9.0, 9.6) | 9.4 (9.0, 9.6) |
| cg14321859 | 5.8 (5.4, 6.0) | 5.9 (5.4, 6.2) | 5.8 (5.4, 6.2) |
| cg03067613 | 8.1 (7.8, 8.4) | 8.1 (7.8, 8.4) | 8.1 (7.8, 8.4) |
| cg11296553 | 0.4 (0.3, 0.4) | 0.4 (0.3, 0.5) | 0.4 (0.3, 0.4) |
| cg16509445 | 8.3 (8.2, 8.5) | 8.3 (8.2, 8.5) | 8.3 (8.2, 8.5) |
| cg16344026 | 1.0 (0.8, 1.2) | 0.9 (0.7, 1.2) | 0.9 (0.8, 1.2) |
| cg15354625 | 8.4 (8.1, 8.5) | 8.3 (8.2, 8.5) | 8.3 (8.2, 8.5) |
| cg23836542 | 1.2 (1.1, 1.4) | 1.2 (1.1, 1.4) | 1.2 (1.1, 1.4) |
| cg07511564 | 1.9 (1.6, 2.2) | 1.9 (1.7, 2.1) | 1.9 (1.6, 2.2) |
| cg18799510 | 1.3 (1.1, 1.5) | 1.3 (1.1, 1.5) | 1.3 (1.1, 1.5) |
| cg14996807 | 8.3 (8.1, 8.5) | 8.4 (8.1, 8.7) | 8.3 (8.1, 8.7) |
| cg18431297 | 6.9 (6.3, 7.3) | 6.8 (6.1, 7.5) | 6.9 (6.3, 7.3) |

^1^ Values are reported as median (Q1, Q3). Degree of methylation was a continuous variable calculated by log-transforming the normalized values and multiplying by 10 to put it on a scale from 0 to 10 (see Methods section).
